# Irinotecan, docetaxel and oxaliplatin combination in metastatic gastric or gastroesophageal junction adenocarcinoma

**DOI:** 10.1038/sj.bjc.6603917

**Published:** 2007-07-31

**Authors:** L Di Lauro, C Nunziata, M G Arena, P Foggi, I Sperduti, M Lopez

**Affiliations:** 1 Division of Medical Oncology B, ‘Regina Elena’ Institute for Cancer Research, Via Elio Chianesi, 53, Rome 00144, Italy; 2 Division of Medical Oncology, San Giuseppe Hospital, Albano Laziale 00041, Italy; 3 Division of Medical Oncology, Toraldo Hospital, Tropea 88038, Italy; 4 Biostatistics Unit, ‘Regina Elena’ Institute for Cancer Research, Rome 00144, Italy

**Keywords:** docetaxel, irinotecan, metastatic gastric cancer, oxaliplatin

## Abstract

This phase II study was designed to evaluate the activity and safety of a combination of irinotecan, docetaxel and oxaliplatin in metastatic gastric or gastroesophageal junction (GEJ) adenocarcinoma. Forty patients with measurable distant metastasis received irinotecan 150 mg m^−2^ and docetaxel 60 mg m^−2^ on day 1, and oxaliplatin 85 mg m^−2^ on day 2. Cycles were repeated every 3 weeks. The primary end point was to demonstrate a 50% improvement in time-to-progression (TTP) over historical controls. All patients were evaluable. Median TTP was 6.5 months (95% confidence interval (CI) 5.6–7.4), the overall response rate was 50% (95% CI 35–65%) and the median overall survival was 11.5 months (95% CI 8.7–14.3). Grade 3/4 neutropaenia occurred in 47.5% of patients. There were four episodes of febrile neutropaenia in three patients. Other non-haematological grade 3 toxicities included diarrhoea in four patients (10%), vomiting in three patients (7.5%) and mucositis in two patients (5%). The irinotecan, docetaxel and oxaliplatin combination chemotherapy is an active and well-tolerated novel regimen for treating metastatic gastric or GEJ adenocarcinoma and deserves further evaluation in randomised trials and in combination with molecular targeting agents.

Gastric cancer is still one of the most prevalent human malignancies, and remains the second leading cause of cancer mortality in the world (Kamangar *et al*, 2006). While the incidence of distal tumours is declining in the Western countries, the incidence of gastroesophageal junction (GEJ) adenocarcinomas, which appear to be more virulent, is rapidly increasing (Devesa and Fraumeni, 1999). The outcome among patients with gastric cancer is determined by the stage of the disease at presentation. Generally, a potentially curative resection is possible in only 30% of all recently diagnosed patients (Hundahl *et al*, 2000), since in the majority of cases the disease is locally advanced or metastatic. Systemic chemotherapy is often used in these patients because randomised trials have shown that combination regimens confer a survival advantage and an improvement in quality-of-life when compared with supportive care alone. Overall, the results have been mostly unsatisfactory with a median time-to-progression (TTP) of 4 months and a median overall survival (OS) of 8–9 months (Wagner *et al*, 2006).

During the last few years, several new drugs including irinotecan (Bleiberg, 1999; Kohne *et al*, 1999), oxaliplatin (Louvet *et al*, 2002; Al-Batran *et al*, 2004), capecitabine (Ajani, 2006) and docetaxel (Thuss-Patience *et al*, 2006) have been demonstrated to be active in metastatic gastric cancer. The incorporation of these agents in new combinations has resulted in regimens such as DCF (docetaxel, cisplatin, fluorouracil), EOX (epirubicin, oxaliplatin, capecitabine) and ILF (irinotecan, leucovorin, fluorouracil), which could have a role in the treatment of these patients but survival is still very poor (Rivera *et al*, 2007). Recently, the combination of irinotecan and oxaliplatin showed activity in advanced gastric cancer with a 50% response rate (RR) and a favourable toxicity profile in 32 patients (Souglakos *et al*, 2004). Furthermore, a synergic effect has been reported with the combination of docetaxel and irinotecan both in untreated and treated patients with gastric cancer (Chang *et al*, 2003; Hawkins *et al*, 2003). It seemed thus appropriate to test the activity and toxicity of three new agents in combination, namely irinotecan, docetaxel and oxaliplatin (IDO), in patients with metastatic gastric or GEJ cancer. These agents have distinct and complementary mechanisms of action, they lack cross-resistance, and they have favourable toxicity profiles. To this purpose, we first performed a phase I study of this combination according to the modified Fibonacci schema. Six patients entered this study, and the recommended phase II doses were as follows: irinotecan 150 mg m^−2^ and docetaxel 60 mg m^−2^ on day 1, oxaliplatin 85 mg m^−2^ on day 2, with cycles repeated every 3 weeks.

## PATIENTS AND METHODS

### Patient selection

Patients with gastric or GEJ adenocarcinoma with distant metastasis not previously treated by systemic chemotherapy were enrolled onto the study. Adjuvant chemotherapy without agents under the study was allowed if completed at least 6 months before. The patients were required to have measurable disease, ECOG performance status ⩽2, life expectancy >3 months, age between 18 and 75 years, adequate bone marrow (absolute neutrophil count ⩾1500 *μ*l^−1^, platelet count ⩾100 000 *μ*l^−1^), renal (serum creatinine ⩽1.5 mg dl^−1^) and liver (serum bilirubin ⩽1.5 mg dl^−1^) functions, normal cardiac function, absence of second primary tumour other than non-melanoma skin cancer or *in situ* cervical carcinoma, no CNS involvement, no previous radiotherapy in parameter lesions, no concurrent uncontrolled medical illness. The protocol was approved and carried out according to the principles of the Declaration of Helsinki and Good Clinical Practice Guidelines, and all patients gave their written informed consent to participate onto the trial.

### Treatment

Treatment consisted of irinotecan 150 mg m^−2^ diluted in 250 ml of normal saline as a 1 h infusion followed by docetaxel 60 mg m^−2^ diluted in 500 ml of normal saline as 1 h infusion on day 1, and oxaliplatin 85 mg m^−2^ diluted in 500 ml 5% dextrose as a 2 h infusion on day 2. Cycles were repeated every 3 weeks. Antiemetic treatment consisted of an antiserotonin agent plus dexamethasone in a 15 min infusion before starting chemotherapy. In addition, orally prednisone premedication was used for prophylaxis of docetaxel-induced hypersensitivity and fluid retention. Atropine 0.25 mg subcutaneously was administered for prophylaxis of cholinergic syndrome. Granulocyte colony-stimulating factor (G-CSF) was used only as secondary prophylaxis once patients had febrile neutropaenia or documented neutropaenic infection. Treatment was postponed by a maximum of 2 weeks if the absolute neutrophil count was less than 1500 *μ*l^−1^ or the platelet count was less than 100 000 *μ*l^−1^. The dose of irinotecan was reduced by 20% of the previous dose in case of grade ⩾3 diarrhoea, whereas oxaliplatin was reduced by 25% in case of grade ⩾2 peripheral neuropathy, and docetaxel by 25% in case of the following toxicities: grade ⩾3 neutropaenia lasting more than 7 days (or in presence of fever), second incidence of febrile neutropaenia despite G-CSF support administered after the first occurrence, grade ⩾3 diarrhoea and grade ⩾3 stomatitis.

Chemotherapy was generally administered on an outpatient basis for a maximum of eight cycles and was discontinued in case of unacceptable toxicity, treatment delay longer than 2 weeks, disease progression or patients' refusal.

### Pretreatment and follow-up studies

Pretreatment evaluation included clinical history and physical examination, automated blood cell count, biochemical profile, ECG and computed tomography of thorax and abdomen. Endoscopy was performed only in case of complete remission of all measurable lesions. Blood counts were obtained weekly; biochemical profile was repeated every 3 weeks. All measurable parameters of disease were re-evaluated every 6 weeks, until the tumour progressed.

### Evaluation of response and toxicity

Patients were evaluated for response to chemotherapy every two cycles of treatment. Responses were assessed by at least two observers, and were confirmed by an expert independent radiologist. The RECIST criteria were used to evaluate clinical response (Therasse *et al*, 2000), and all objective responses were confirmed by CT scans at least 4 weeks after the initial documentation of response. Time-to-progression and OS were calculated from the date of first chemotherapy cycle to the date of disease progression, death or last follow-up evaluation, respectively. Toxicity was assessed in each treatment cycle using the National Cancer Institute Common Toxicity Criteria (version 3.0).

### Statistical considerations

The primary end point of this study was to test the hypothesis that TTP will improve by 50%. The major secondary end point was RR. Other secondary end points were OS and safety. The study was designed to have an 80% power to show an improvement in median TTP from 4 to 6 months with a 5% type I error, two sides, assuming exponential progression-free survival times. A sample size of 40 patients was required to meet these requirements (A'Hern, 2001). Time-to-progression and OS were analysed according to the Kaplan–Meier method, and were updated to 30 April 2007.

## RESULTS

### Patients' characteristics

From February 2004 to April 2006, 40 patients with metastatic gastric or GEJ cancer entered onto this multicentre study. All patients were evaluable for efficacy and toxicity. The pretreatment characteristics of patients are listed in Table 1. None of the patients had previously received chemotherapy for advanced disease; six patients had received adjuvant chemotherapy without irinotecan, docetaxel or oxaliplatin several months before they entered this study (median, 14 months; range, 9–23 months).

### Efficacy

**Figure 1:**
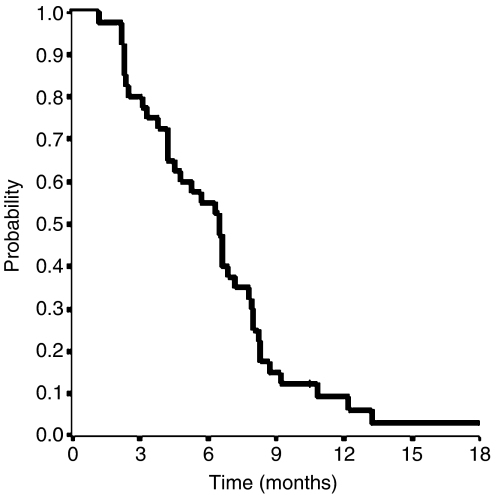
Median TTP for all patients.

**Figure 2:**
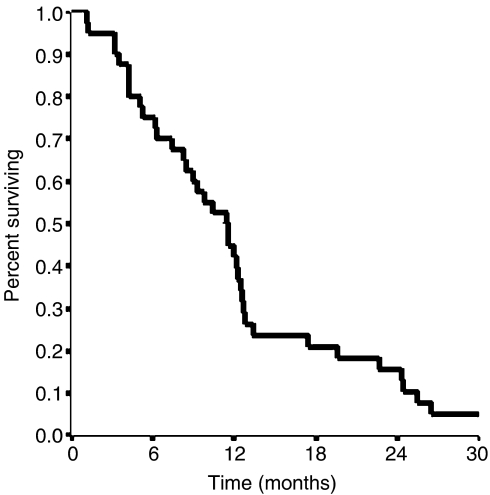
Overall survival for all patients.

Median TTP was 6.5 months (95% confidence interval (CI) 5.6–7.4) (Figure 1). Only six patients progressed within the first 2 months, whereas at the time of this analysis all but one patient progressed. Among 40 assessable patients, we observed two (5%) complete responses (CRs) and 18 (45%) partial responses (PRs), for an overall RR of 50% (95% CI 35–65%). Disease remained stable in 14 (35%) patients (Table 2). Responses according to predominant site of disease, were as follows: liver 16 of 28 patients (57%); nodes/peritoneum 3 of 10 patients (30%); lung 1 of 2 patients. RRs did not significantly differ according to number of metastatic sites: one site, 7 of 12 patients (58%); two sites, 9 of 18 patients (50%); and three or more sites, 4 of 10 patients (40%). Responses were seen in 3 of 6 patients (50%) who received adjuvant chemotherapy and in 17 of 34 patients (50%) not previously treated with chemotherapy. Responses were observed also in 13 of 26 patients (50%) with primary tumour not resected and in 7 of 14 patients (50%) with primary tumour resected. Response rates and TTP did not differ when patients were evaluated according to the primary site of disease (gastric: 50% and 6.5 months; GEJ: 50% and 6.3 months, respectively). Upon disease progression, 21 patients (52.5%) received a second-line chemotherapy, including epirubicin/fluorouracil (*n*=16) and cisplatin/capecitabine (*n*=5). Median OS was 11.5 months (95% CI 8.7–14.3 months) (Figure 2). One- and two-year survivals were 42 and 15.8%, respectively. Thirty-eight patients had died at the time of the present evaluation. Twenty-three of the 32 patients (72%) who had tumour-related symptoms before therapy showed an improvement in at least one of their symptoms without worsening of any other symptom.

### Toxicity

Haematological toxicity data are listed in Table 3. A total of 242 cycles of IDO regimen were analysed in 40 patients, with a median of seven cycles administered per patient (range, 1–8 cycles). The most important toxicity was myelosuppression, which occurred almost always on day 8 (docetaxel nadir). Grade 3 and 4 neutropaenia were recorded in 40 and in 7.5% of the patients, respectively. Four episodes of febrile neutropaenia occurred in 3 (7.5%) patients. In these patients, a 25% dose reduction of docetaxel was required, whereas treatment was postponed in two (5%) patients and in six (2.5%) cycles because of a delay in bone marrow recovery. Mean irinotecan, docetaxel and oxaliplatin dose intensities were 45.45, 18.36 and 27.17 mg m^−2^ per week, respectively, which are equivalent at 90.9, 91.8 and 95.9% of the planned dose intensities for these drugs. Grade 3 thrombocytopaenia was observed in 2.5% of the patients, and grade 3 anaemia occurred in 10% of the patients.

Non-haematological toxicities are listed in Table 4. Mild-to-moderate diarrhoea, which was reversible and manageable in all cases, occurred in 40% of the patients, while grade 3 diarrhoea developed in four (10%) patients. Alopecia was frequent. Mild nausea and vomiting was encountered in 45% of the patients, and was severe in three (7.5%) patients. Mild and transient peripheral neurotoxicity was recorded in 35% of the patients. Hypersensitivity reactions, which not precluded chemotherapy continuation, were recorded in 5% of the patients. No cardiotoxicity or treatment-related deaths were observed.

## DISCUSSION

Classical cisplatin-based regimens obtain responses in 20–40% of the patients with metastatic gastric or GEJ cancer, a median TTP of about 4 months and a median survival of 8–9 months (Wagner *et al*, 2006). Despite recent advances in the chemotherapy of this disease, the survival advantage appears to be marginal, and no regimens have emerged that could be considered standard.

To develop a potentially more effective front-line chemotherapy, combination regimens have generally included one or two of the three new drugs active in gastric cancer, namely irinotecan, docetaxel and oxaliplatin. This is the first study that combines these three agents.

The IDO regimen appears to be an active treatment in previously untreated patients with metastatic gastric cancer, since the primary end point of this study was largely achieved with a median TTP of 6.5 months. Noteworthy are also the overall RR of 50% and the median survival of 11.5 months. These data appear of particular importance considering that liver involvement and primary tumour unresected were recorded in 70 and 65% of the patients, respectively.

These results compare favourably with those reported in several trials using new generation treatment regimens. In a recent phase III randomised trial, the combination of docetaxel, cisplatin and fluorouracil demonstrated significant superiority in comparison to the reference regimen of cisplatin and fluorouracil (CF). Nevertheless, TTP, OS and RR were 5.6 months, 9.2 months and 37%, respectively (Van Cutsem *et al*, 2006). In another phase III study of 337 patients with advanced gastric cancer, irinotecan in combination with bolus fluorouracil and infused fluorouracil over 22 h did not result in statistically significant improvement in TTP (5.0 *vs* 4.2 months) or OS (9.0 *vs* 8.7 months) compared with CF, but showed a better safety profile (Dank *et al*, 2005). Moreover, our results are similar to those observed with the combination of EOX in the REAL-2 phase III study. In this trial, the EOX regimen showed RR of 48% and OS of 11.2 months in 239 patients with advanced oesophageal, EGJ or gastric carcinoma with a manageable toxicity (Cunningham *et al*, 2006).

The combination used in the current study compares favourably also with regimens including two of these three new agents. In two phase II studies in advanced gastric cancer, combined irinotecan and oxaliplatin yielded a median TTP of 5.5 and 5.3 months, and an OS of 8.5 and 9.5 months (Souglakos *et al*, 2004; Woell *et al*, 2006). When irinotecan was used in combination with docetaxel in 90 untreated patients with advanced gastric cancer enrolled in two phase II studies, a median TTP of 3.8 and 4.5 months with an OS of 8.2 and 9.0 months were observed (Hawkins *et al*, 2003; Park *et al*, 2006). Treatment results do not seem to improve by combining oxaliplatin and docetaxel, since TTP and OS were 4.4 and 9.5 months, respectively, in 71 patients with stage IV gastroesophageal and/or stomach cancer (Richards *et al*, 2006).

Of considerable importance in patients with metastatic gastric cancer are treatment tolerance and quality-of-life, since these patients have generally symptoms at baseline. The IDO regimen demonstrated a tolerable toxicity profile in the present trial. The most important side effect was grade 3–4 neutropaenia, which occurred in less than 50% of the patients, and almost always at docetaxel nadir. Grade 3 diarrhoea was observed in four patients (10%). Other severe toxic effects were infrequent, as were dose reductions and treatment delays. Although quality-of-life was not measured in our study, the majority of patients enjoyed a clear clinical benefit during treatment, as evidenced by the relief of pain and other tumour-related symptoms. Treatment compliance was good, as demonstrated by relative dose intensities of 90.9% for irinotecan, 91.8% for docetaxel and 95.8% for oxaliplatin. Moreover, chemotherapy was generally administered on an outpatient basis.

In conclusion, we found that the combination of irinotecan, docetaxel and oxaliplatin is active with an acceptable safety profile in the first-line treatment of metastatic gastric or GEJ adenocarcinoma, and should be compared with other active regimens (e.g. ECF and EOX) in a randomised fashion. However, in order to further improve treatment results in this disease, the addition of targeted therapy to cytotoxic agents should be considered. In two recent phase II trials, the combination of irinotecan, cisplatin and bevacizumab (Shah *et al*, 2006), and cetuximab combined with FOLFIRI (Pinto *et al*, 2007), both produced encouraging results with TTP and OS remarkably improved over historical controls. In this context, the IDO regimen, with its activity and safety profile, could be a good candidate to enhance survival in metastatic gastric or GEJ cancer patients.

## Figures and Tables

**Table 1:** Patient's characteristics

**Characteristic**	**No. of patients**	**%**
Patients evaluable	40	100
		
*Age (years)*		
Median	62	
Range	38–75	
		
*Sex*		
Male	28	70
Female	12	30
		
*ECOG PS*		
0	8	20
1	26	65
2	6	15
		
*Disease location*		
Gastric	30	75
GEJ	10	25
		
*Histologic type*		
Diffuse	18	45
Intestinal	16	40
Unspecified	6	15
		
Previous adjuvant chemotherapy	6	15
		
*Status of primary tumour*		
Unresected	26	65
Resected	14	35
		
*Predominat site of disease*		
Liver	28	70
Nodes/peritoneum	10	25
Lung	2	5
		
*No. of metastatic sites*		
1	12	30
2	18	45
⩾3	10	25
		
*Symptoms*		
Weight loss		
No	13	32.5
⩽10%	16	40
>10%	11	27.5
		
*Anorexia*		
No	21	52.5
Yes	19	47.5
		
*Dysphagia*		
No	23	57.5
Yes	17	42.5
		
*Pain*		
No	20	50
Yes	20	50

Abbreviations: ECOG PS=Eastern Cooperative Oncology Group Performance Status; GEJ=gastroesophageal junction.

**Table 2:** Objective response in 40 patients

**Response**	**No. of patients**	**%**
Complete response	2	5
Partial response	18	45
Stable disease	14	35
Progressive disease	6	15

**Table 3:** Grade 3/4 haematological toxicity per cycle and per patient

	**% of 242 cycles**		**% of 40 patients**	
**Toxicity**	**Grade 3**	**Grade 4**	**Grade 3**	**Grade 4**
Neutropaenia	26	5	40	7.5
Thrombocytopaenia	1	—	2.5	—
Anaemia	4.5	—	10	—

Four episodes of febrile neutropaenia in three patients.

**Table 4:** Non-haematological toxicity in 40 patients

**Toxicity**	**Grade 1 %**	**Grade 2 %**	**Grade 3 %**
Nausea/vomiting	25	20	7.5
Mucositis	20	15	5
Diarrhoea	20	20	10
Fatigue	25	15	7.5
Fluid retention^a^	15	10	—
Alopecia	30	45	25
Neurotoxicity	20	15	—
Hypersensitivity reaction	2.5	2.5	—

a Grade 1–2=mild; grade 3=severe.
